# Oral Bioavailability and Plasma Disposition of Pefloxacin in Healthy Broiler Chickens

**DOI:** 10.3389/fvets.2017.00077

**Published:** 2017-05-24

**Authors:** María-Aránzazu Martínez, Irma Ares, José-Luis Rodríguez, Marta Martínez, María-Rosa Martínez-Larrañaga, Gerardo Isea, Arturo Anadón

**Affiliations:** ^1^Department of Toxicology and Pharmacology, Faculty of Veterinary Medicine, Universidad Complutense de Madrid, Madrid, Spain

**Keywords:** pefloxacin, broiler chickens, IV and oral dose, kinetics, PK/PD modeling

## Abstract

The pharmacokinetics of pefloxacin after single 10 mg/kg BW intravenous (IV) and oral doses were studied in healthy broiler chickens. For 24 h, serial blood samples were obtained after IV and oral administration. Concentrations of pefloxacin and its major metabolite *N*-demethyl pefloxacin (norfloxacin) were measured by use of high-performance liquid chromatography. The plasma concentrations–time data were found to fit a two-compartment open model. For pefloxacin, the elimination half-life (*t*_½β_) was 8.44 ± 0.48 and 13.18 ± 0.82 h after IV and oral administration, respectively. After single oral dose, pefloxacin was rapidly absorbed with an absorption half-life (*t*_½a_) and *T*_MAX_ of 0.87 ± 0.07 and 2.01 ± 0.12 h, respectively. Maximum plasma concentration (*C*_MAX_) was 4.02 ± 0.31 µg/mL. Oral bioavailability of pefloxacin was found to be 70 ± 2%. Pefloxacin was converted to *N*-demethyl pefloxacin (norfloxacin). This metabolite represented 5% of the parent drug plasma concentrations. The maximal plasma concentration (*C*_MAX_) of *N*-demethyl pefloxacin (norfloxacin) was calculated as 0.19 ± 0.01 mg/mL. The *t*_½β_ of *N*-demethyl pefloxacin after oral pefloxacin administration was 10.93 ± 0.80 h. The results indicate that an oral dose of 10 mg pefloxacin/kg BW, every 24 h, should be effective in treatment of the most systemic infections in poultry.

## Introduction

Pefloxacin [1-ethyl-6-fluoro-1-4-dihydro-4-oxo-7(4-methyl-1-piperazinyl) quinolone-3 carboxylic acid] is a fluorinated quinolone, which is structurally related to nalidixic acid. Pefloxacin has high antibacterial activity against Gram-negative bacteria including the most species of Enterobacteriaceae and *Neisseria, Campylobacter, Haemophilus* species, and Gram-positive bacteria such as *Staphylococcus*, and *Streptococcus*, between others (1). Antibacterial active structural analogs of nalidixic acid inhibit prokaryotic DNA gyrase *in vitro*. Pefloxacin and other 4-quinolones inhibit DNA gyrase activity and DNA replication. DNA gyrase inserts negative superhelical turns into bacterial DNA and belongs to a group of enzymes known as DNA topoisomerases that are responsible for controlling the spatial geometry of DNA *in vivo*. Inhibiting the action of DNA gyrase prevents the supercoiling and relaxation of DNA. The bactericidal effect of the 4-quinolones probably results from the inhibition of the resealing of open nicks in the DNA strand produced by DNA gyrase (1, 2).

**Figure 1:**
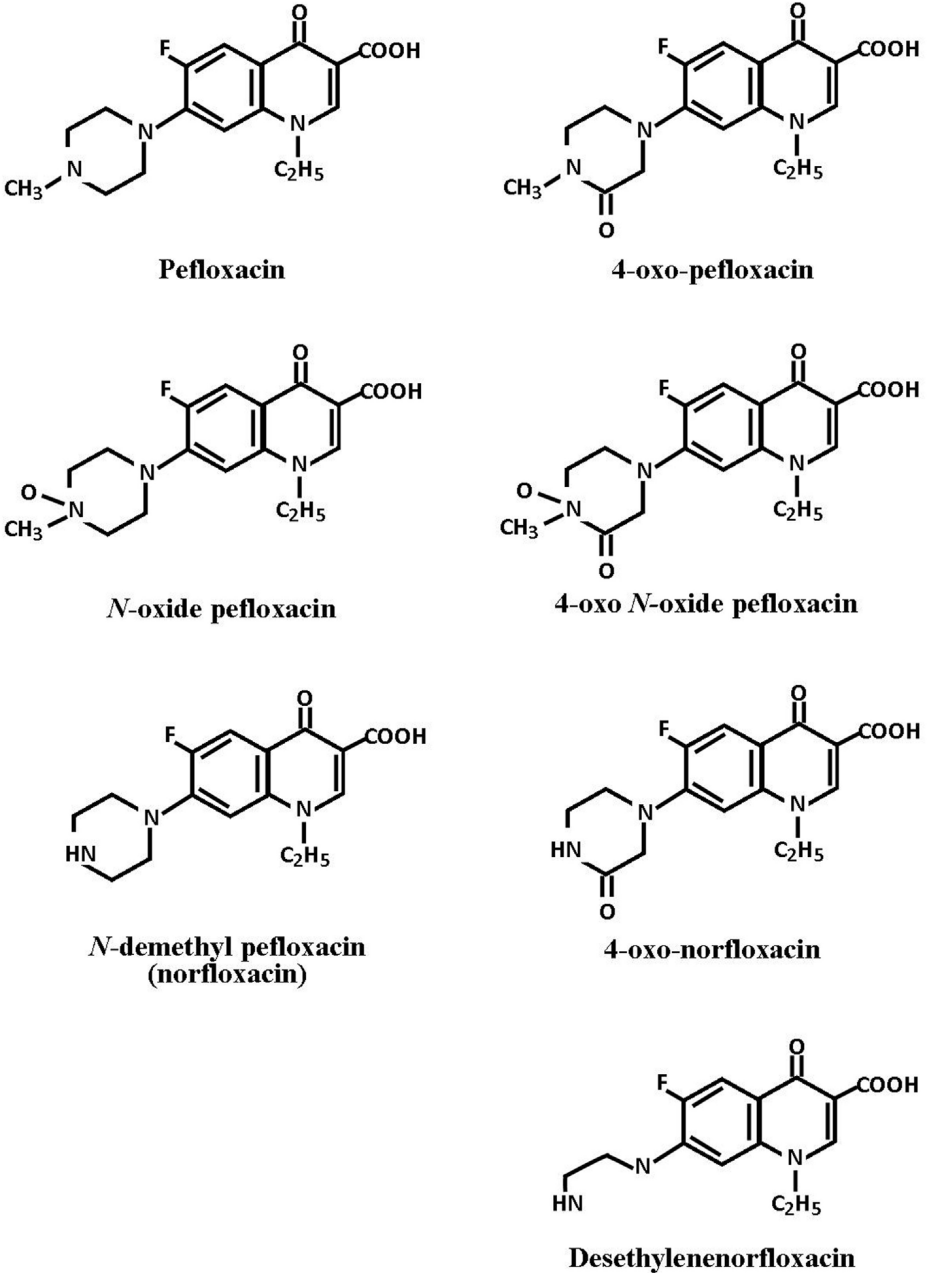
**Chemical structures of pefloxacin and metabolites**.

A large number of structurally related analogs of nalidixic acid have been developed. In particular, the 4-quinolones such as ciprofloxacin, enoxacin, fleroxacin, lomefloxacin, and ofloxacin have broad spectrums of antibacterial activity, are rapidly bactericidal, penetrate most body fluids and tissues, and have demonstrated promising results in the treatment of a variety of infectious conditions (3–6). Pefloxacin is another 4-quinolone, which has similar *in vitro* antibacterial activity against most Gram-negative and Gram-positive bacteria. It is rapidly absorbed following oral administration, achieving steady state serum concentrations that are in excess of the MIC values for most pathogens. In addition, pefloxacin is extensively distributed throughout the body, producing high tissue concentrations. Because of its broad spectrum activity, it is likely that pefloxacin should have potential therapeutic application for many types of systemic infections. Pefloxacin has been shown to be generally well tolerated during short- and long-term oral administration. The pefloxacin metabolism is extensive (85–90%). The piperazinyl ring is the main site of metabolism. The ring is hydroxylated, N-oxidized, demethylated, formylated, and acetylated. Six metabolites of pefloxacin have been identified (Figure 1); the major metabolites are *N*-demethyl pefloxacin (norfloxacin) and pefloxacin *N*-oxide; the latter has low antibacterial activity (7). In poultry medicine, the potential usefulness of pefloxacin for treatment of common infections in chickens for fattening requires detailed information on pharmacokinetics properties to establish the orally administered dose necessary for maintaining bactericidal drug concentrations in the body. Although studies on the kinetic behavior of pefloxacin in many species including man (8–10), calves (11), rabbits (12), goats (13, 14), pigs (15), and sheep (16) are available; pharmacokinetic investigations on pefloxacin in poultry are limited (17, 18). Results of a preliminary study in our laboratory in chickens were presented as a poster at 9th International EAVPT Congress (19). The objective of this study was to determine the pharmacokinetics of pefloxacin after IV and oral administration in broiler chickens.

## Materials and Methods

### Chemicals and Reagents

Pefloxacin mesylate dihydrate (P0106 Sigma) (≥97% purity) and its metabolite *N*-demethyl pefloxacin (norfloxacin) (Y0001301 Sigma-Aldrich) analytical standard (≥98% purity) were provided by Sigma-Aldrich, Spain. All other chemicals used were obtained from usual commercial sources and were of the highest grade available.

### Animals

All experimental procedures involving animals were reviewed in accordance with ethics requirements and authorized by the official ethical committee of the Universidad Complutense de Madrid. Eighteen healthy Ross male chickens for fattening that were 40-days old and that each weighed 2.5 kg were used. The chickens, purchased at a poultry breeding farm (Nutreco, S.A., Sada Division, Cazalegas, Toledo, Spain) were placed individually in cages in the animal house of our university 1 week before the start of the study. Clinical signs of disease were not apparent. The animal house was maintained at 25 ± 2°C and 45–65% relative humidity as previously described (20). Antibiotic free commercial feed and water were available *ad libitum*.

### Experimental Design

Eighteen birds were allotted to three groups. Chickens in groups A and B (eight broiler chickens/group) were given single IV or oral doses of pefloxacin (10 mg/kg BW). Chickens of group C (*n* = 2) did not receive any treatment and were used to determine the validation criteria of the analytical method. For IV administration, 500 mg of pefloxacin was dissolved in 10 mL water (sterilized 0.9% saline solution) to give a stock solution of 50 mg/mL (0.5 mL of stock solution is given IV for each animal of 2.5 kg BW, equivalent to 10 mg/kg BW). For oral gavage administration, 100 mg of pefloxacin was dissolved in 10 mL water (sterilized 0.9% saline solution) to give a stock solution of 10 mg/mL (2.5 mL of stock solution is given orally for each animal of 2.5 kg BW, equivalent to 10 mg/kg BW). Pefloxacin was administered IV into the right brachial vein of chickens in group A or orally directly into the crop using a thin plastic tube attached to a syringe. Food but not water was withheld for 12 h before oral dosing.

Blood samples (1 mL) were drawn from all chickens *via* cannula from the left brachial vein into heparinized syringes at 0.16, 0.33, 0.5, 1, 2, 4, 6, 8, 12, and 24 h after drug administration. Plasma was separated after centrifugation (1,500 *g* for 10 min) and was stored frozen at −45°C until assayed for pefloxacin and *N*-demethyl pefloxacin (norfloxacin) concentrations; experimental design previously described (21).

### Analytical Method and Validation

Pefloxacin and *N*-demethyl pefloxacin (norfloxacin) concentrations in plasma were measured using an HPLC technique (22) with modifications.

#### Plasma Extraction

Plasma samples were separately extracted in methylene chloride as described (23, 24). The plasma sample was added to 8 mL of methylene chloride and 0.5 mL of 0.5 M sodium phosphate buffer pH 7.5. The tube was mechanically shaken and centrifuged at 2,500 *g* for 10 min. The organic phase (lower layer) was transferred into other disposable tube. This extraction was repeated three times, and all organic phases were pooled. Sodium hydroxide (0.5 M, 0.5 mL) was then added to the plasma extract, and the tube was shaken at 1,500 *g* for 10 min. The aqueous phase (upper layer) was collected and frozen (−45°C) until HPLC assay. A 20 µL aliquot was injected into the HPLC column for assay of pefloxacin and *N*-demethyl pefloxacin (norfloxacin).

#### HPLC Analysis

Plasma concentrations of pefloxacin and *N*-demethyl pefloxacin (norfloxacin) were determined using a Shimadzu liquid chromatographic system equipped with a system controller CBM-20A/CBM-20 Alite, two solvent delivery modules LC-20AD, a spectrofluorimetric detector RF-10A_XL_, and LC solution software. All samples were analyzed using a 5 µm particle size Nucleosil C18 column (12.5 cm × 0.4 cm) preceded by a C18 guard column. The mobile phase (pH 4.8) was acetonitrile (150 mL), sodium acetate trihydrate (2 g), citric acid monohydrate (2 g), trimethylamine (1 mL), and water (850 mL), and a flow rate of 2 mL/min was used. The excitation and emission wavelengths of the detector were 330 and 440 nm, respectively. Peak areas in the sample chromatograms were quantitated by external standard technique using solutions of pefloxacin and norfloxacin reference standards.

The analytical method was fully validated according to EU requirements for the compounds pefloxacin and *N*-demethyl pefloxacin (norfloxacin) norfloxacin [linearity, recovery rate, accuracy, precision, trueness, quantification limit (LOQ), detection limit (LOD) and specificity] (25). Drug concentrations were determined from peak areas and the use of calibration curves obtained by running plasma samples from broiler chickens not administered pefloxacin (i.e., chickens of group C) that were fortified with pefloxacin as well as with *N*-demethyl pefloxacin (norfloxacin). For plasma samples as determined by use of the linear least squares regression procedure, a linear relationship existed in the calibration curve of pefloxacin and *N*-demethyl pefloxacin over the range of 0.01–20 µg/mL, which always yielded a correlation coefficient exceeding 0.9998. Overall mean recovery of pefloxacin and *N*-demethyl pefloxacin (norfloxacin) from plasma was greater than 96%. Within-day and day-to-day precision were <5.5%. The LOQ was 0.02 µg/mL for pefloxacin and 0.03 µg/mL for *N*-demethyl pefloxacin (norfloxacin) in the plasma. Interference of endogenous compounds was verified on blank plasma from untreated chickens, which provided the specificity of the method. This method differs from those reported by Pant et al. (17) and Dimitrova et al. (18) who used, respectively, chloroform-isopentanol, acetonitrile or only protein precipitation with perchloric acid, instead of methylene chloride for the extraction of pefloxacin and *N*-demethyl pefloxacin (norfloxacin) from plasma.

#### Data Analysis

Plasma concentration *versus* time data was sequentially fitted to 1-, 2- and multiple-compartment models, using the computer program Phoenix (Version 7.0; Pharsight Corporation, Mountain View, CA, USA). The model was determined for best fit on the basis of a smaller value for the Akaike information criterion (26). The two-compartment model was the best fit for all broiler chickens. This model was used to establish pharmacokinetic parameters as described for other drugs (20, 21, 27). Plasma curves of pefloxacin after a single IV and oral administration and those of *N*-demethyl pefloxacin (norfloxacin) (the main metabolite in plasma) after a single IV and oral administration of pefloxacin were obtained for each chicken and were fitted to the following exponential equations (20, 21, 27):

$$C=A_{1}\;e^{-\alpha t}+\;A_{2}\;e^{-}{\;}^{\beta t}\left(\text{IV}\right)$$

$$C=A_{1}\;e^{-\alpha t}+A_{2}\;e^{-}{\;}^{\beta t}-\;A_{\text{3}}\;e^{-K_{\text{a}}t}\left(\text{oral}\right)$$

where *C* is the plasma concentration of drug; *A*_1_, *A*_2_, and *A*_3_ are mathematical coefficients (i.e., *A*_1_ and *A*_2_ are the plasma concentrations extrapolated to time 0 of the first and second elimination phases of drug and *A*_3_ for the absorption phase); α is the hybrid rate constant for the distribution phase; β is the hybrid rate constant for the elimination terminal phase (i.e., α and β are the slopes of the first and second elimination phases of the drug disposition); and *K*_a_ the first-order absorption rate constant and *t* is the time. Absorption half-life (*t*_½a_), half-life of α phase (*t*_½α_), half-life of β phase (*t*_½β_), distribution rate constants for transfer of the drug from the central to the peripheral compartment (*K*_12_) and from the peripheral to the central compartment (*K*_21_), and the elimination rate constant (*K*_10_) were calculated using standard equations as described (28, 29). After IV and oral administration, the area under the concentration–time curves (AUC) was calculated as follows:

$$\begin{array}{l} \text{AUC}=(A_{1}/\alpha )+(A_{2}/\beta );\text{ or}\\ \text{AUC}=(A_{1}/\alpha )+(A_{2}/\beta )\;-\text{ (}A_{3}/K_{\text{a}}\text{)} \end{array}$$

Total plasma clearance (CL) was calculated, using the following formula:

CL=(dose/kg)/AUC; or CL=(dose/kg)  (F/AUC)

Oral bioavailability (*F*) was determined as follows:

$$F=({\text{AUC}}_{\text{oral}})/({\text{AUC}}_{\text{IV}})$$

Oral bioavailability (*F*) was calculated from the ratio between the value of AUC_oral_ for each chicken and the mean value of AUC_IV_ for the eight chickens used in the IV administration study. Complete absorption was determined on the basis of AUC_IV_, which represents the mean AUC for the eight broiler chickens to which pefloxacin was administered. Because of the small individual variation in AUC_IV_ and the fact that the same eight chickens were not available for oral and IV studies, the mean AUC_IV_ rather than AUC_IV_ for each chicken was used to estimate bioavailability after oral administration of pefloxacin.

Mean residence time (MRT) was calculated as follows:

$$\text{MRT}=(A_{1}/\alpha^{2}+A_{2}/\beta^{2})\;\times \text{ (}1/\text{AUC)}$$

Apparent volume of distribution [*V*_d(area)_] was determined as follows:

$$V_{\text{d}(\text{area})}=(\text{dose/kg})\text{/AUC}\cdot \beta ;\text{ or }V_{\text{d(area)}}=(\text{dose/kg})(F)\text{/AUC}\cdot \beta$$

Volume of distribution at steady state (*V*_ss_) was determined as follows:

$$V_{\text{ss}}\text{=MRT}\times \text{CL}$$

Maximum drug plasma concentration (*C*_MAX_) after oral administration and the time at which *C*_MAX_ was achieved (*T*_MAX_) was determined directly from the concentration *versus* time curve.

Mean pharmacokinetic variables were obtained by averaging the variables calculated for drug disposition after each pefloxacin administration in each broiler chicken.

Differences in pharmacokinetic data between dosing routes were analyzed for statistical significance by the Mann–Whitney *U* test. Differences of *P* < 0.05 were considered significant. All data were tabulated as mean ± SD.

## Results

### Plasma Pefloxacin Disposition after Single IV and Oral Administration

**Figure 2:**
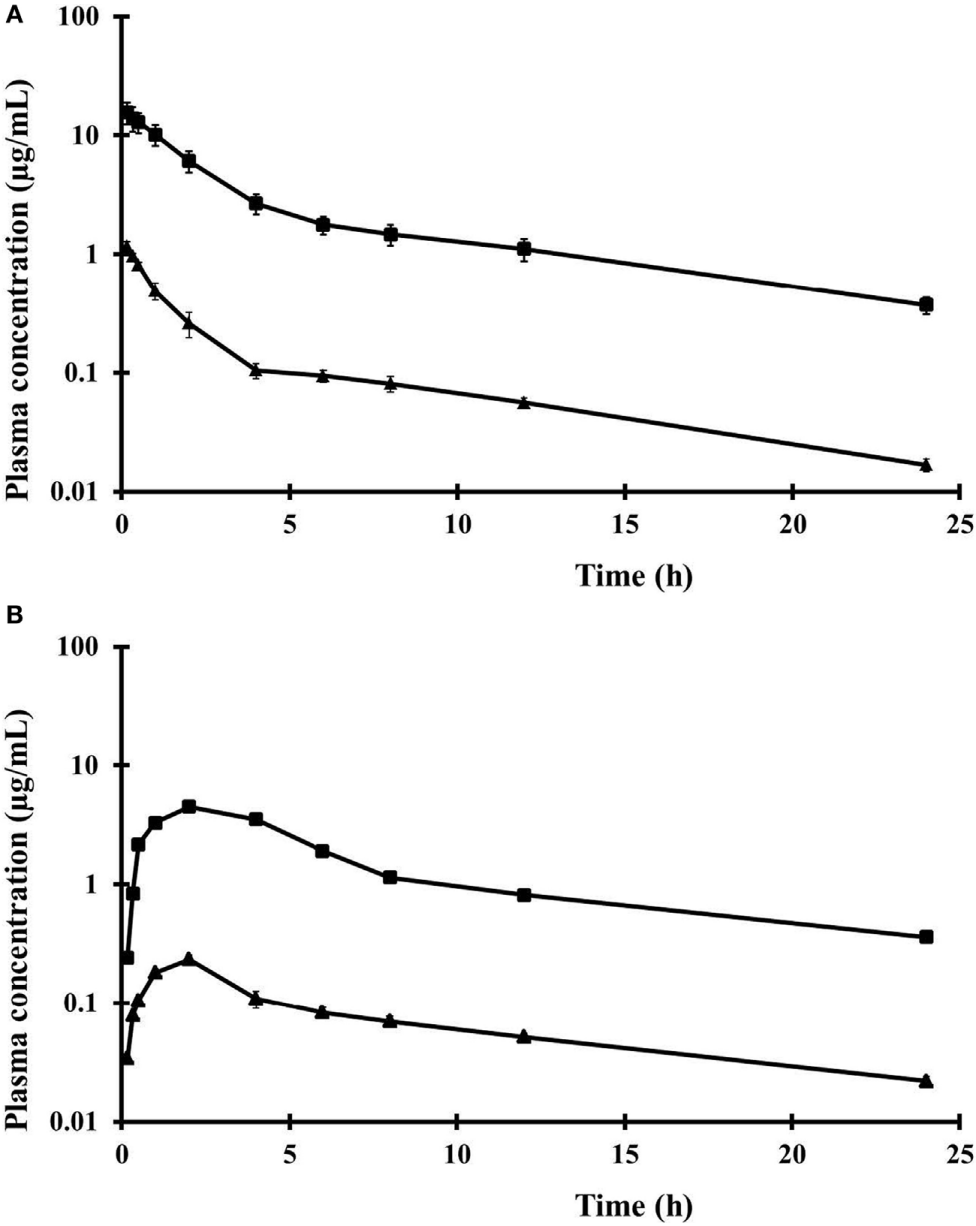
**Plasma concentrations of pefloxacin (■) and *N*-demethyl pefloxacin (norfloxacin) (▲) after single IV administration (A) and after single oral administration (B) of 10 mg pefloxacin/kg BW**. Data are expressed as mean ± SD values for eight broiler chickens. Symbols without bars indicate that the SD is within the symbols.

Mean plasma concentrations (in micrograms per milliliter, ±SD) of pefloxacin and its metabolite *N*-demetil pefloxacin (norfloxacin) obtained after single oral and IV dose of pefloxacin are presented in Figure 2. The plasma concentration–time profile of pefloxacin and *N*-demetil pefloxacin after oral and IV administration of pefloxacin for each chicken were similar to the overall means. Values of the parameters that described absorption and disposition kinetics of pefloxacin in broiler chickens are presented in Table 1. The kinetic parameters of the metabolite *N*-demetil pefloxacin (norfloxacin) after oral administration of pefloxacin are summarized in Table 2.

**Table 1:** **Pefloxacin kinetic parameters for broiler chickens after a single IV and oral administration of pefloxacin (10 mg/kg BW)**.

Parameter	IV	Oral
*A*_1_ (μg/mL)	14.77 ± 3.50	16.33 ± 5.13
*A*_2_ (μg/mL)	2.69 ± 0.63	1.27 ± 0.16
*A*_3_ (μg/mL)	–	18.40 ± 5.33
α (h^−1^)	0.70 ± 0.06	0.43 ± 0.07
β (h^−1^)	0.082 ± 0.005	0.054 ± 0.004
*K*_a_ (h^−1^)	–	0.80 ± 0.07
*t*_½α_ (h)	1.00 ± 0.09	1.68 ± 0.36
*t*_½β_ (h)	8.44 ± 0.48	13.18 ± 0.82***
*t*_½a_ (h)	–	0.87 ± 0.07
*V*_d(area)_ (L/kg)	2.36 ± 0.46	3.55 ± 0.21
*V*_ss_ (L/kg)	1.54 ± 0.33	–
*K*_12_ (h^−1^)	0.28 ± 0.03	0.16 ± 0.04
*K*_21_ (h^−1^)	0.18 ± 0.02	0.10 ± 0.01
*K*_10_ (h^−1^)	0.32 ± 0.04	0.21 ± 0.03
AUC (mg/h/L)	53.56 ± 10.09	37.71 ± 0.96
*F* (%)	–	70 ± 2
MRT (h)	7.94 ± 0.50	13.57 ± 0.72
CL (L/h/kg)	0.19 ± 0.04	0.19 ± 0.004
*C*_MAX_ (μg/mL)	–	4.02 ± 0.31
*T*_MAX_ (h)	–	2.01 ± 0.12

Values are the mean ± SD, n = 8. Significantly different ***P < 0.001.

*A_1_, *A*_2_, and *A*_3_, mathematical coefficients; α, hybrid rate constant for distribution phase; β, hybrid rate constant for terminal elimination phase. *K*_a_, first-order absorption rate constant; *t*_½a_, absorption half-life; *t*_½α_, half-life at α phase; *t*_½β_, half-life at β phase; *V*_d(area)_, apparent volume of distribution; *V*_ss_, volume of distribution at steady state; *K*_12_, distribution rate constant for transferring the drug from the central to the peripheral compartment; *K*_21_, distribution rate for transfer from peripheral to central compartment; *K*_10_, elimination rate constant; AUC, area under the concentration–time curve; MRT, mean residence time; CL, total plasma clearance; *C*_MAX_, maximal concentration in plasma after oral administration; *T*_MAX_, time needed to reach *C*_MAX_*.

*–, Not applicable*.

**Table 2:** ***N*-demethyl pefloxacin (norfloxacin) kinetic parameters for broiler chickens after a single IV and oral administration of the parent drug, pefloxacin (10 mg/kg BW)**.

Parameter	IV	Oral
*T*_½α_ (h)	0.60 ± 0.20	1.25 ± 0.13
*t*_½β_ (h)	8.02 ± 0.47	10.93 ± 0.80
*K*_12_ (h^−1^)	0.61 ± 0.19	0.24 ± 0.05
*K*_21_ (h^−1^)	0.21 ± 0.03	0.21 ± 0.02
*K*_10_ (h^−1^)	0.51 ± 0.07	0.17 ± 0.01
AUC (mg/h/L)	2.70 ± 0.33	1.93 ± 0.18
MRT (h)	7.54 ± 0.47	13.77 ± 0.86
*C*_MAX_ (μg/mL)	–	0.19 ± 0.01
*T*_MAX_ (h)	–	1.51 ± 0.12

*Values are the mean ± SD, n = 8*.

*See Table 1 for abbreviations*.

After IV administration of pefloxacin, a rapid distribution phase (*t*_½α_ = 1.00 ± 0.09 h) and a slower elimination phase (*t*_½β_ = 8.44 ± 0.48 h) were observed (Table 1). The apparent volume of distribution [*V*_d(area)_] and at steady state (*V*_ss_) and clearance (CL) values were 2.36 ± 0.46 L/kg, 1.54 ± 0.33 L/kg, and 0.19 ± 0.04 L/h/kg, respectively (Table 1). Pefloxacin was rapidly and widely absorbed after oral administration (10 mg/kg BW). Pefloxacin concentrations in plasma 10 and 20 min were 0.24 ± 0.02 and 0.84 ± 0.02 µg/mL, and plasma drug concentrations were 1.15 ± 0.03 and 0.81 ± 0.02 µg/mL for 8 and 12 h, respectively. The half-life of oral absorption (*t*_½a_) was 0.87 ± 0.07 h. Bioavailability (*F*) of pefloxacin after oral administration was 70 ± 2%. Maximal plasma concentration of pefloxacin (*C*_MAX_: 4.02 ± 0.31 µg/mL) was detected at 2.01 ± 0.12 h after oral administration.

A fraction of pefloxacin was biotransformed to *N*-demethyl pefloxacin (norfloxacin) after oral and IV administration of pefloxacin. This metabolite represented 5% of the parent drug plasma concentrations, as calculated by the ratio between the mean AUC for *N*-demetil pefloxacin and mean AUC for pefloxacin after oral and IV administration of pefloxacin. Plasma concentration of *N*-demethyl pefloxacin (0.19 ± 0.01 µg/mL) peaked at 1.51 ± 0.12 h after oral administration of pefloxacin. The *t*_½β_ of *N*-demethyl pefloxacin after oral pefloxacin administration was 10.93 ± 0.80 h (Table 2).

### Efficacy Predictors

Table 3 shows the estimated values for AUC_24_/MIC and *C*_MAX_/MIC for the MIC90 (upper value) and MIC50 (lower value) (1, 30–33). The applied *C*_MAX_ and AUC_24_ values for single oral administration were 4.02 µg/mL and 37.71 mg/h/L, respectively.

**Table 3:** **Efficacy predictors (*C*_MAX_/MIC and AUC_24_/MIC) estimated for pefloxacin against susceptible bacteria in broiler chickens after a single oral dose of 10 mg/kg BW**.

Bacteria	*C*_MAX_/MIC	AUC_24_/MIC (h)
*Escherichia coli*—MIC 0.25–0.06 (μg/mL)	16.08–67	150.84–628.5
*Salmonella* spp.—MIC 0.25–0.03 (μg/mL)	16.08–134	150.84–1,257
*Pseudomonas aeruginosa*—MIC 2–0.06 (μg/mL)	2.01–67	18.85–628.5
*Haemophilus influenza*—MIC 0.06–0.03 (μg/mL)	67–134	628.5–1,257
*Shigella* spp.—MIC 0.2–0.06 (μg/mL)	20.1–67	188.55–628.5
*Staphylococcus aureus*—MIC 0.50–0.25 (μg/mL)	8.04–16.08	75.42–150.84
*Mycoplasma gallisepticum*—MIC 0.1 (μg/mL)	40.2	377.1

*For calculations, the MIC applied values were MIC_90_ upper and MIC_50_ lower (26–30)*.

## Discussion

The present paper is the first to describe the kinetic parameters for pefloxacin and its metabolite *N*-demethyl pefloxacin (norfloxacin) in chickens for fattening. The other major metabolite *N*-oxide pefloxacin was not investigated because it is practically inactive (7). In this study, the kinetics of pefloxacin and *N*-demethyl pefloxacin (norfloxacin) after a single IV and oral administration of pefloxacin (10 mg/kg BW) were determined. Disposition of pefloxacin and *N*-demethyl pefloxacin (norfloxacin) after IV and oral administration of pefloxacin in chickens was best described by use of a two-compartment model, in accordance with most of the results reported for humans (8, 10), calves (11), goats (13, 14), pigs (15), and sheep (16), but not for ducks and chickens (17, 18).

The kinetic variables obtained after IV administration showed for pefloxacin a rapid distribution (*t*_½α_ = 1.00 ± 0.09 h) and a slow elimination phase (*t*_½β_ = 8.44 ± 0.48 h). Pefloxacin is well distributed to the tissues [*V*_d(area)_ = 2.36 ± L/kg and *V*_ss_ = 1.54 ± 0.33 L/kg, respectively]. The elimination half-life of pefloxacin after IV administration (*t*_½β_ = 8.44 ± 0.48 h) was much longer than those previously reported in other studies: 2.21 h in calves (10), 1.12–1.6 h in goats (12, 13), and 2.84–3.25 in ducks (18) but comparable to that reported in sheep (*t*_½β_ = 6.88 h) (16).

Pefloxacin was rapidly (*t*_½a_ = 0.87 ± 0.07 h) and extensively absorbed after oral administration with a comparable maximal plasma concentration (*C*_MAX_ = 4.02 ± 0.31 µg/mL) but a shorter *T*_MAX_ (2.01 ± 0.12 h) to those values previously reported in the literature for broiler chickens (*C*_MAX_ = 3.78 ± 0.23 µg/mL at a *T*_MAX_ = 3.33 ± 0.21 h) (17). However, the *C*_MAX_ and *T*_MAX_ detected in our study for pefloxacin were both much higher than those reported for ducks (*C*_MAX_ = 1.42 ± 0.20 µg/mL at a *T*_MAX_ = 1.35 ± 0.23 h) (18). Oral bioavailability of pefloxacin was 70 ± 2% in our study, which was comparable to that reported in ducks (60–68%) (18). In the present study, plasma concentrations (0.84 ± 0.02 µg/mL) were achieved in a relatively short time (20 min) and maintained up to 12 h (1.15 and 0.81 µg/mL), equivalent values previously observed (18). In our study, *t*_½β_ (13.18 ± 0.82 h) after oral dose of pefloxacin was statistically higher than the *t*_½β_ (8.44 ± 0.48 h) after IV administration. This difference may be the result of continued absorption of pefloxacin probably by an enterohepatic circulation with reabsorption of the drug from the gastrointestinal tract.

Pefloxacin was poorly metabolized in chickens by *N*-demethylation. In the present study, in broiler chickens the metabolite *N*-demethyl pefloxacin (norfloxacin) only represented 5% of the parent drug plasma concentrations, showing that this metabolite with a lower potency of activity than parent compound (7) should not be taken into account to calculate antimicrobial dosing regimen of pefloxacin for broiler chickens. The maximal plasma concentration (*C*_MAX_) of *N*-demethyl pefloxacin (norfloxacin) was calculated as 0.19 ± 0.01 mg/mL, value much lower than that previously reported in broiler chickens (*C*_MAX_: 0.80 ± 0.07 mg/mL) (17). The small value of *C*_MAX_ also indicates that in chickens the antibiotic action can mostly be attributed to the parental drug pefloxacin rather than its metabolite of *N*-demethyl pefloxacin (norfloxacin). These results of the present study are comparable to those previously reported in dog, monkey, and humans after oral doses of pefloxacin (8). The antimicrobial activity in dog, monkey, and human plasma was largely due to the parent drug which, respectively, accounted for 64, 94, and 84% of the total activity (8).

In the present study, the rate of elimination of *N*-demethyl pefloxacin (norfloxacin) (*t*_½β_: 10.93 ± 0.80 h) after oral pefloxacin administration was more rapid than that of pefloxacin (*t*_½β_: 13.18 ± 0.82 h) (*P* < 0.01) but not after IV pefloxacin administration (*t*_½β_ of *N*-demethyl pefloxacin: 8.02 ± 0.47 h and *t*_½β_ of pefloxacin: 8.44 ± 0.48). In a previous study in chickens, the elimination half-life of *N*-demethyl pefloxacin (norfloxacin) after oral dose of 10 mg pefloxacin/kg BW was 5.66 h (17). This difference is probably the result of the different kinetic profile (two-compartment open model) applied in our study for the metabolite norfloxacin.

The integration of pharmacokinetic–pharmacodynamic (PK–PD) data represents the most approach to determining dosing regimens of antimicrobial drugs for subsequent evaluation in disease models and clinical trials (34, 35). As fluoroquinolones, against most if not all susceptible pathogens, kill bacteria by a concentration-dependent killing action, the PK–PD variables widely used to predict effective doses are the ratio of the AUC at 24 h to the MIC (AUC_24h_/MIC) and the ratio of the maximal drug concentration to the MIC (*C*_MAX_/MIC) (36). The AUC_24h_/MIC ratio is the most important variable in predicting such effects, with the rate of clinical cure being greater than 80% when this ratio is higher that 100–125 h (37–39). The second predictor of efficacy for concentration-dependent antibiotic is the ratio *C*_MAX_/MIC, considering that values above 8–10 would lead to better clinical results, as well as, to prevent resistant bacterial mutants surviving treatment (38, 40, 41). Nevertheless, it must be emphasized that these values provide general guidance, and lower or higher numerical values may apply against organisms of all classes (36). In the present study, considering the AUC_24h_/MIC and *C*_MAX_/MIC ratios obtained (Table 3), it can be concluded that pefloxacin administered orally at a dose rate of 10 mg/kg BW in broiler chickens might be effective against bacteria with MIC values ≤0.50 µg/mL but not sufficient for treating infections of bacteria with MIC over 0.8 µg/mL. Nevertheless, further investigation on pefloxacin plasma disposition in broiler chickens after multiple oral doses (10 mg pefloxacin/kg BW, daily for 5 days) should be carried out to recommend that oral dose rates of 10 mg/kg p.c. once daily produce blood concentrations that should be effective against susceptible bacteria in broiler chickens.

## Ethics Statement

The study was undertaken in accordance with the ethics requirements and authorized by the official ethical committee for animal testing of our university.

## Author Contributions

The authors have contributed in the paper to fix the objectives based on the related works reported in the literature, apply methodology, and calculate the results and discuss them.

## Conflict of Interest Statement

The authors declare that the research was conducted in the absence of any commercial or financial relationships that could be construed as a potential conflict of interest.
